# The impact of sedation and analgesia scores on prognosis in critically ill sepsis patients with sepsis-associated encephalopathy: a retrospective analysis

**DOI:** 10.3389/fneur.2025.1622964

**Published:** 2025-06-25

**Authors:** Weiqin Wei, Donghai Fang, Xiaochun Hu, Yongfang Zhou, Jiangquan Fu, Guofeng Wu

**Affiliations:** ^1^The Second Affiliated Hospital of Suzhou University, Suzhou, Jiangsu, China; ^2^Department of Emergency, Affiliated Hospital of Guizhou Medical University, Guiyang, Guizhou, China

**Keywords:** sedation and analgesia score, sepsis-associated encephalopathy, clinical efficacy, prognosis, sepsis

## Abstract

**Background:**

Sepsis is a critical condition resulting from a poor immune response to infection, often leading to complications like sepsis-associated encephalopathy (SAE). Research suggests a link between sedation and analgesia use and SAE development in intensive care unit (ICU) patients, but study inconsistencies limit definitive conclusions. This study aims to explore the relationship between sedation and analgesia scores and the occurrence of SAE in the ICU, as well as their impact on clinical effectiveness and patient prognosis.

**Methods:**

Between January 1, 2021, and August 30, 2022, a retrospective analysis of 356 sepsis cases was conducted in the Emergency ICU of the Affiliated Hospital of Guizhou Medical University. After excluding 102 patients, 219 were included and divided into SAE and non-SAE groups for analysis.

**Results:**

The SAE group demonstrated higher age, Sequential Organ Failure Assessment (SOFA) scores, and APACHE II scores, alongside longer ICU durations and lower Glasgow Coma Scale (GCS) scores (*p* < 0.05) compared to the non-SAE group. Furthermore, the levels of lactate dehydrogenase (LDH), interleukin-6 (IL-6), and blood lactate were significantly increased in the SAE group (*p* < 0.05). After adjustments for baseline characteristics, biochemical indices, risk assessment scores, and clinical features, multivariate analysis identified age, APACHE II score, LDH, IL-6, oxygenation index, base excess (BE), and base excess of extracellular fluid (BE(ecf)) as significant risk factors for encephalopathy in septic patients (*p* < 0.05). ROC curve analysis indicated that the area under the curve (AUC) for predicting SAE was 0.810 (95% CI: 0.785–0.831) for the APACHE II score, 0.780 (95% CI: 0.743–0.801) for IL-6, and 0.769 (95% CI: 0.730–0.836) for BE. Sensitivity values were 81.1, 77.4, and 70.6%, while specificity values were 70.3, 72.3, and 71.3%. Patients with sepsis influenced by these factors exhibited an increased likelihood of developing SAE. Additionally, RASS and BPS scores were significantly correlated with the prognosis of sepsis patients (*p* < 0.05).

**Conclusion:**

The study demonstrated that patients with SAE exhibit physiological disturbances, including elevated inflammatory markers (IL-6 and LDH), impaired oxygenation, and acid–base imbalances, which may contribute to more severe clinical courses. Additionally, RASS and BPS scores were found to be reliable indicators of patient prognosis in sepsis. These findings may guide clinical practice in managing patients with SAE.

## 1 Introduction

Deficiencies of the immune system lead to sepsis, a life-threatening condition caused by the dysfunctional response of the host to infection. In the United States, about 750,000 people suffer from sepsis every year, of which 20–30% die from sepsis-related complications (1, 2). In 2017, there were 48,900,000 cases of sepsis worldwide, and 11 million people died from sepsis, accounting for 19.7% of the total number of deaths globally (3). It is among the top three contributors to in-hospital mortality (4). Sepsis is a significant threat to patient health and causes a considerable economic and social burden. Therefore, investigating the mechanisms of sepsis is an important way to understand and explore its treatment. The imbalance of the inflammatory response caused by sepsis is a significant factor contributing to septic injury, often leading to dysfunction in multiple organs. Among these, sepsis-associated encephalopathy (SAE) is a manifestation of central nervous system impairment in patients with sepsis (5, 6). Currently, the exact pathogenesis of SAE remains unknown. However, possible underlying mechanisms include blood–brain barrier damage, neuronal apoptosis and autophagy, neuroinflammation, neurotransmitter imbalance, vascular endothelial damage, oxidative stress (7), complement activation, and immunosuppression (8). The synergistic interaction of these factors may contribute to the development of SAE, with the activation of one factor potentially triggering others. Additionally, SAE may induce progressive immunosuppression, leading to uncontrollable infections by activating the sympathetic nervous system and the hypothalamic–pituitary–adrenal axis.

SAE has been identified as an independent risk factor for mortality in septic patients, according to statistical research. Additionally, studies have shown that patients with SAE experience a worse long-term prognosis compared to those with non-SAE, including the potential for persistent nervous system dysfunction (9, 10). However, the current challenge lies in the absence of standardized diagnostic criteria for SAE. While there is a generally accepted definition that refers to alterations in consciousness among sepsis patients, after excluding factors such as central nervous system infection, inadequate perfusion, structural brain injury, and metabolic causes, the diagnosis primarily relies on detecting subtle impairments in attention, orientation, and handwriting ability. Consequently, the diagnosis remains largely exclusive and constrained. Therefore, investigating the relevant high-risk factors associated with SAE could not only aid in diagnosing patients but also establish a foundation for early intervention in managing this condition.

With advancements in medical care, the mortality rate from sepsis has significantly decreased. However, due to the complex nature of the disease and its low diagnostic rates, there remains a gap in the prevention and treatment of neurological dysfunction in sepsis patients. For survivors, neurological impairment is the most disabling long-term consequence, severely affecting their quality of life and leading to substantial economic and social costs. The World Health Organization (WHO) has recognized it as a key focus for health prevention and management efforts (11). Patients who survive sepsis usually experience cognitive and behavioral disorders after discharge, with a high incidence of mental illness. Various factors contribute to SAE and can lead to serious adverse outcomes. Therefore, early diagnosis and appropriate intervention for SAE can reduce mortality and disability rates, as well as improve rehabilitation outcomes for survivors. The exact etiology of SAE, however, remains unclear. Previous studies (12, 13) have identified a correlation between sedation and analgesia scores and the development of intensive care unit (ICU) SAE. Sedation and analgesia therapy have been shown to improve clinical outcomes and prognosis in critically ill patients (14). However, consistent and convincing conclusions have not yet been established due to variations in trial design, sample selection criteria, and observation indicators. Therefore, further in-depth research is necessary. This retrospective study aims to comprehensively investigate the relationship between sedation and analgesia scores and the development of SAE in ICU patients. The findings of this study will contribute to developing more effective diagnostic, intervention, and prevention strategies for SAE in patients with sepsis.

## 2 Patients and methods

### 2.1 General information

A total of 356 sepsis cases from January 1, 2021, to August 30, 2022, in the Emergency ICU of the Affiliated Hospital of Guizhou Medical University were retrospectively analyzed. Of these, 102 patients who did not meet the admission criteria were excluded, resulting in 219 patients included in the study. SAE was defined as cerebral dysfunction occurring in the presence of sepsis that met the inclusion criteria. Our hospital’s ethics committee approved the study, and the general data of the patients is presented in Table 1. The inclusion criteria were: (a) All selected cases met the relevant diagnostic criteria for sepsis, as defined by the Sepsis-3 criteria (15). According to the Sepsis-3 criteria (15), the diagnosis of SAE requires the presence of sepsis (evidenced by infection and organ dysfunction) alongside altered mental status, while excluding other potential causes or alternative diagnoses that could account for the changes in mental status; (b) all patients in the SAE group satisfied the relevant diagnostic criteria for SAE and were confirmed through clinical examination (16).

**Table 1:** Baseline characteristics of patients in the SAE group and the non-SAE group.

Grouping	SAE group (*n* = 105)	Non-SAE group (*n* = 114)	*t/χ^2^*	*p*
Age (years)	77.61 ± 14.75	62.13 ± 15.35	7.597	<0.001
Gender (*n*/%)			2.980	0.084
Male	70 (66.67)	63 (55.26)		
Female	33 (33.33)	51 (44.74)		
BMI(kg/m^2^)	23.55 ± 4.55	23.61 ± 4.60	0.097	0.923
Number of years of education (years)	9.33 ± 1.20	9.38 ± 1.24	0.303	0.762
ICU stay time (days)	15.89 ± 3.38	10.33 ± 3.20	12.504	<0.001
Mean arterial pressure(mmHg)	75.89 ± 21.33	78.12 ± 20.55	0.788	0.432
GCS score (points)	9.52 ± 3.76	15.00 ± 3.27	11.531	<0.001
APACHE II score(points)	23.79 ± 9.07	15.04 ± 3.93	9.389	<0.001
SOFA score(points)	4.50 ± 2.11	2.70 ± 1.32	7.648	<0.001
28-day death rate (%)	51 (48.57)	48 (42.11)	0.923	0.337
Infection site			2.210	0.027
Lungs	46 (43.81)	28 (24.56)		
Abdominal cavity	30 (28.57)	45 (39.47)		
Urinary system	23 (21.90)	35 (30.70)		
Skin or soft tissue	3 (2.86)	1 (0.88)		
Blood	4 (3.81)	5 (4.39)		

Values are expressed as mean ± standard deviation or *n* (%). APACHE II score, Acute Physiology and Chronic Health Evaluation II score; BMI, Body Mass Index; GCS, Glasgow Coma Scale; ICU, Intensive Care Unit; SAE, Sepsis-Associated Encephalopathy; SOFA, Sequential Organ Failure Assessment.

Exclusion criteria included: (a) individuals under 18 years of age; (b) pregnant or lactating individuals; (c) individuals with congenital brain dysplasia; (d) individuals with primary brain injuries, such as traumatic brain injury, intracranial infections, cerebral infarction, cerebral hemorrhage, or epilepsy; (e) individuals with secondary encephalopathy, such as hepatic, pulmonary, or uremic encephalopathy, or those who underwent cardiopulmonary resuscitation. The sample size for this study was calculated using the following formula for comparing two proportions:


$$n_{1}={\frac{\left[Z_{\alpha /2}\sqrt{p(1-p)(1+c)/c}+Z_{\beta }\sqrt{p_{1}(1-p_{1})+p_{2}(1-p_{2})/c}\right]}{{\left(p_{1}-p_{2}\right)}^{2}}}^{2}$$

**Figure 1:**
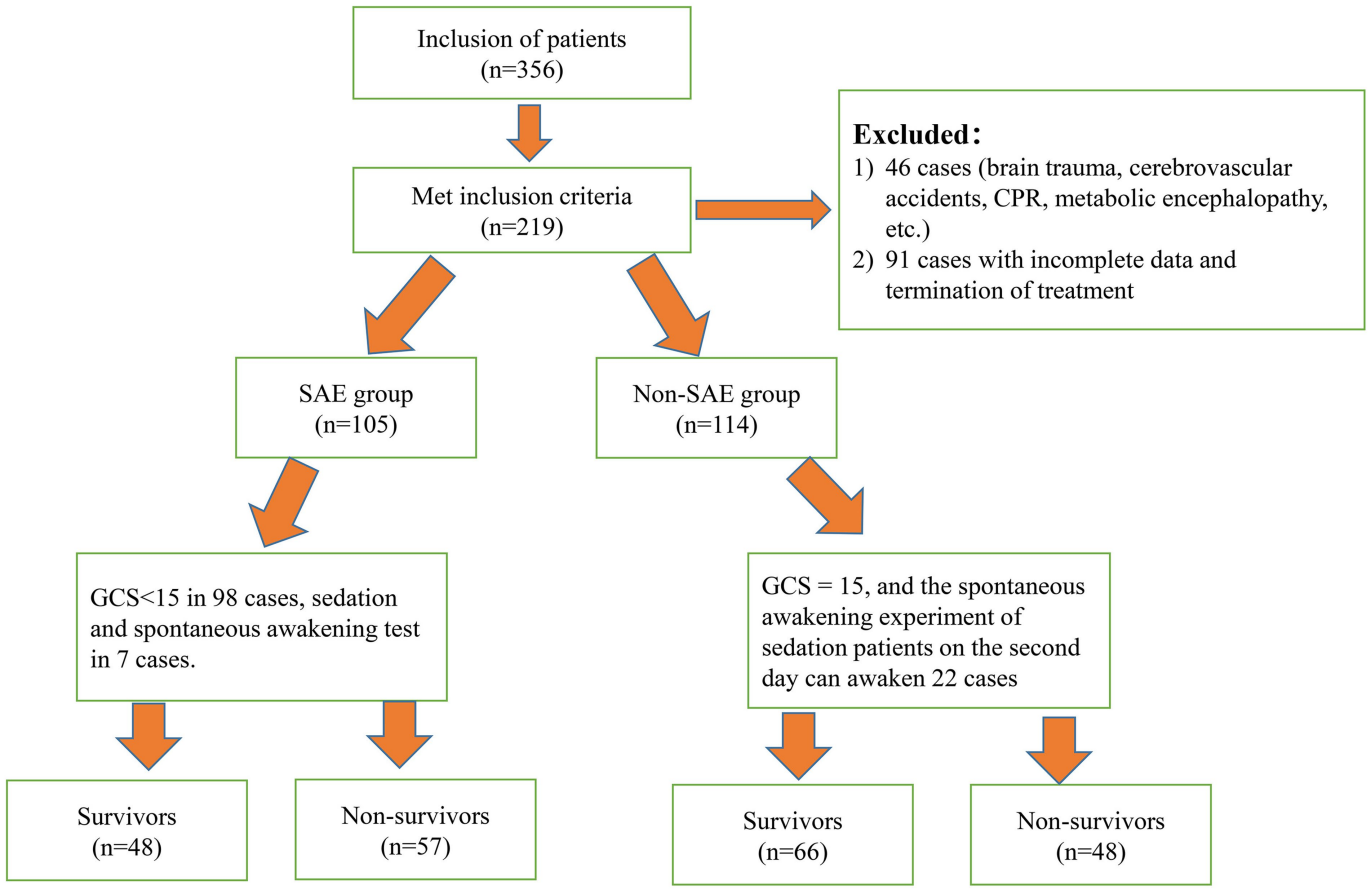
Technology roadmap of the study.

The parameters were set as follows: a bilateral alpha (*α*) level of 0.05, a beta (*β*) level of 0.20, P1 = 0.95, P2 = 0.75, and c = 1 (indicating equal group sizes). The incidence of sepsis-related encephalopathy in patients with sepsis was taken as the effect index, and the related literature and previous studies were reviewed (17). After calculation, the total sample size was determined to be 199 cases, and 219 patients were included based on a 10% attrition rate. The detailed technology roadmap of the study is presented in Figure 1.

### 2.2 Data collection and treatment methods

All patients were hospitalized, and their general clinical and laboratory data were collected from the Electronic Health Records system of our hospital. The general clinical data gathered in this study included information on age, sex, previous health status, presence of shock, and site of infection. Additionally, clinical data obtained within 24 h after admission to the ICU included the APACHE II score (18) and the GCS score (19). Laboratory data were collected within the first 24 h after ICU admission. Laboratory data comprised a complete blood count (CBC) with parameters such as WBC, hemoglobin (Hb), hematocrit (HCT), and platelet count (PLT). Coagulation tests, including the International Normalized Ratio (INR), prothrombin time (PT), activated partial thromboplastin time (APTT), and fibrinogen (FIB) levels, were also analyzed. Furthermore, biochemical tests included urea, creatinine, total bilirubin, alanine aminotransferase (ALT), aspartate aminotransferase (AST), creatine kinase (CK), creatine kinase-MB (CKMB), lactate dehydrogenase (LDH), blood glucose, sodium, potassium, calcium, procalcitonin (PCT), and interleukin-6 (IL-6). The parameters collected included pH, base excess (BE), base excess of extracellular fluid (BE(ecf)), and lactic acid. In addition to the duration of ICU hospitalization, 28-day mortality was also recorded. Based on the occurrence of SAE, patients were classified into the SAE group or the non-SAE group (patients with sepsis but no encephalopathy). The correlation between sedation and analgesia scores and ICU SAE was analyzed using logistic regression analysis. The biochemical indices required for SOFA and APACHE II scoring were collected from the patient’s peripheral venous blood serum and measured by our hospital’s biochemical laboratory using a complete set of analyzers (Roche C702, Roche, Switzerland). PCT, IL-6, and CRP were measured using serum from peripheral venous blood with a chemiluminometer (Roche E411, Roche, Switzerland). Coagulation function was assessed using plasma from peripheral venous blood with a fully automatic coagulation analyzer (Max-4, STA R Max). Routine blood tests were conducted using whole blood from peripheral venous blood and measured with a fully automatic blood cell analyzer (XN-9000, Sysmex). Blood gas analysis was performed by drawing arterial blood from the patient and analyzing it with a blood gas analyzer (GEM Premier 5,000, Werfen). The diagnostic criteria for SAE were based on relevant literature (20). All patients received the same sedation and analgesia treatment. The RASS sedation scale (21) and the BPS (22) scores were used to evaluate the sedation and analgesia effects of the two groups.

### 2.3 Observation index

Sedation and analgesia scores were assessed by trained ICU nursing staff following standardized hospital protocols to ensure consistency and reliability. Although multiple clinicians were involved, all received uniform training, and inter-rater reliability was periodically assessed as part of routine quality assurance.

#### 2.3.1 Richmond agitation-sedation scale (RASS)

The RASS was utilized for assessing a patient’s level of sedation or agitation. The RASS was administered at regular intervals, typically every 2 hours, according to ICU protocols. Sedation targets were individualized based on clinical status, with protocols designed to prevent both over-sedation and under-sedation, guided by multidisciplinary team evaluations. The scale ranges from +4 to −5, with specific descriptions for each level: +4 points: aggressive and violent; +3 points: very restless, trying to pull out the breathing tube, stomach tube, or intravenous drip; +2 points: agitation and anxiety, intense body movement, unable to cooperate with the ventilator; +1 point: anxiety, with progress but only slight body movement; 0 points: sober and calm, in a natural state; −1 point: sleepy, not fully awake but can stay awake for more than 10 s; −2 points: mild sedation, unable to stay awake for more than 10 s; −3 points: moderate sedation, responsive to sound; −4 points: severe sedation, responding to physical stimulation; −5 points: coma, with no response to physical or verbal stimulation.

#### 2.3.2 The behavioral pain scale (BPS)

BPS assessments were conducted for all mechanically ventilated patients, in accordance with ICU pain management guidelines. Non-intubated patients were evaluated using alternative validated pain scales appropriate to their clinical condition; BPS was not applied to these patients. For subjective indicators such as vocalization, clinicians adhered to standardized criteria outlined in the BPS manual to minimize variability. Sedation and analgesia management adhered to established institutional guidelines consistent with national standards. The BPS consists of three items: facial expression, upper limb movement, and ventilation compliance (for intubated patients) or vocalization (for non-intubated patients), with each item scored from 1 to 4. The total score ranges from 3 to 12 points, with higher scores indicating greater pain in patients.

### 2.4 Statistical analysis

The data analysis was conducted using SPSS22.0 statistical software. The measurement indicators with normal distribution and uniform variance are presented by (x̄±s), and independent sample *t*-tests were adopted to compare groups. The metrological index of non-normal distribution was expressed by median / quartile, the Mann–Whitney test compared groups, the counting index was expressed by [*n* (%)], and the χ^2^ test was carried out. Pearson correlation analysis was used to analyze the correlation between RASS score, BPS score, and prognosis of sepsis patients. Logistic multivariate regression analysis was adopted to identify the risk factors of sepsis-related encephalopathy. APACHE II score, LDH, IL-6, oxygenation index, and the predictive ability of BE and BE(ecf) to the occurrence of SAE were evaluated by ROC. All *p*-values were calculated using two-tailed tests, with statistical significance defined as *p* < 0.05.

## 3 Results

### 3.1 Baseline characteristics of patients

In total, 105 patients were assigned to the SAE group and 114 patients to the non-SAE group (patients with sepsis without encephalopathy). Compared to the non-SAE group, the SAE group had higher age, Sequential Organ Failure Assessment (SOFA) scores, and APACHE II scores; longer ICU stay times; and lower GCS scores (*p* < 0.05). The two groups had no significant differences regarding sex, height, body mass index, years of education, average arterial pressure, 28-day mortality rate, or infection site (*p* > 0.05). All results are presented in Table 1.

### 3.2 Comparison of biochemical indexes

Significant differences were observed in WBC, platelet count (PLT), lactate dehydrogenase (LDH), interleukin-6 (IL-6), oxygenation index, base excess (BE), BE(ecf), and lactate (Lac) levels, with the SAE group showing worse values for these parameters (all *p* < 0.05). Other markers such as hemoglobin (Hb), hematocrit (HCT), creatinine (Cr), glucose, and inflammatory markers like procalcitonin (PCT) showed no significant differences between the groups (all *p* > 0.05). All results are presented in Table 2.

**Table 2:** Comparison of biochemical indexes between SAE and Non-SAE groups.

Laboratory index	SAE group (*n* = 105)	Non-SAE group (*n* = 114)	*t/χ^2^*	*p*
WBC (×10^9^/L)	10.99 ± 2.21	12.39 ± 2.45	4.427	<0.001
N (%)	84.50 ± 4.56	84.88 ± 5.31	0.566	0.572
Hb(g/L)	105.00 ± 6.34	104.90 ± 4.23	0.138	0.890
HCT	0.28 ± 0.53	0.29 ± 0.54	0.138	0.890
PLT(×10^9^/L)	101.40 ± 12.11	108.90 ± 12.45	4.512	<0.001
INR	2.89 ± 0.65	2.77 ± 0.69	1.322	0.188
PT(sec)	55.40 ± 5.56	54.90 ± 5.78	0.651	0.516
APTT(sec)	55.40 ± 5.23	54.91 ± 5.11	0.701	0.484
FIB(g/L)	3.60 ± 0.42	3.64 ± 1.21	0.321	0.748
D-D(ng/mL)	2610.00 ± 153.42	2612.00 ± 155.56	0.096	0.924
Total protein(g/L)	53.07 ± 10.96	57.25 ± 11.57	1.429	0.155
Albumin(g/L)	28.56 ± 6.52	29.57 ± 7.59	1.052	0.294
UREA(mmol/L)	5.50 ± 1.21	5.56 ± 1.43	0.334	0.739
Cr(μmol/L)	215.40 ± 12.21	218.55 ± 12.66	1.871	0.063
TBIL(μmol/L)	33.30 ± 5.23	33.20 ± 3.12	0.173	0.863
ALT(U/L)	41.50 ± 5.53	41.49 ± 5.67	0.013	0.990
AST(U/L)	77.00 ± 3.12	77.85 ± 4.64	1.577	0.116
CK(U/L)	674.50 ± 123.54	678.80 ± 125.85	0.255	0.799
CKMB(U/L)	215.70 ± 5.77	216.80 ± 5.89	1.394	0.164
LDH(U/L)	215.70 ± 5.67	256.00 ± 5.88	51.544	<0.001
Glucose(mmol/L)	8.55 ± 2.54	8.33 ± 2.11	0.699	0.485
Na(mmol/L)	134.00 ± 5.23	135.30 ± 5.77	1.742	0.083
K(mmol/L)	3.92 ± 1.12	3.84 ± 1.22	0.504	0.615
Ca(mmol/L)	2.10 ± 0.53	2.17 ± 0.75	0.791	0.430
PCT(ng/L)	45.48 ± 38.68	50.62 ± 36.75	1.008	0.314
IL-6(pg/L)	928.71 ± 163.74	528.71 ± 176.51	17.344	<0.001
Oxygenation index	165.34 ± 56.28	198.24 ± 46.28	4.740	<0.001
PH	7.35 ± 1.24	7.37 ± 1.13	0.125	0.901
BE(mmol/L)	1.20 ± 0.29	1.01 ± 0.33	4.510	<0.001
BE(ecf)(mmol/L)	0.92 ± 8.21	1.02 ± 6.33	2.650	0.009
Lac(mmol/L)	2.80 ± 1.61	2.10 ± 1.78	3.043	0.003

Values are expressed as mean ± standard deviation or *n* (%). ALT, Alanine Aminotransferase; APTT, Activated Partial Thromboplastin Time; BE, Base Excess; CK, Creatine Kinase; CKMB, Creatine Kinase-MB; D-D, D-Dimer; BE(ecf), Base excess of extracellular fluid; FIB, Fibrinogen; Hb, Hemoglobin; HCT, Hematocrit; IL-6, Interleukin-6; INR, International Normalized Ratio, K, Potassium; Lac, Lactic Acid; LDH, Lactate Dehydrogenase; Na, Sodium; PCT, Procalcitonin; PLT, Platelet Count; PT, Prothrombin Time; TBIL, Total Bilirubin; WBC, White Blood Cells.

### 3.3 Multivariate analysis of factors affecting sepsis-related encephalopathy

After adjusting for baseline characteristics, biochemical indices, risk assessment scores, and clinical characteristics, multivariate analysis showed several significant risk factors for SAE in ICU patients. These included age (OR = 1.672, 95% CI: 1.241–2.252), APACHE II score (OR = 1.557, 95% CI: 1.114–2.177), LDH (OR = 1.534, 95% CI: 1.108–2.124), IL-6 (OR = 1.517, 95% CI: 1.144–2.012), oxygenation index (OR = 1.738, 95% CI:1.084–2.788), base excess (OR = 1.772, 95% CI:1.017–3.085), and BE(ecf)(OR = 1.756, 95% CI: 1.078–2.861). In contrast, SOFA scores, WBC count, platelets, and lactate levels were not significant predictors in this model (Table 3).

**Table 3:** Logical analysis of risk factors for sepsis-related encephalopathy using multivariate logistic regression.

Variable	*b*	S. E	Chi-square value	*p* value	OR	95% CI
Age	0.514	0.152	11.435	0.001	1.672	1.241–2.252
APACHE II	0.443	0.171	6.711	0.010	1.557	1.114–2.177
SOFA	0.241	0.222	1.178	0.278	1.273	0.824–1.966
WBC	0.231	0.260	0.789	0.374	1.260	0.757–2.097
PLT	0.215	0.206	1.089	0.297	1.240	0.828–1.857
LDH	0.428	0.166	6.648	0.010	1.534	1.108–2.124
IL-6	0.417	0.144	8.386	0.004	1.517	1.144–2.012
Oxygenation index	0.553	0.241	5.256	0.022	1.738	1.084–2.788
BE	0.572	0.283	4.085	0.043	1.772	1.017–3.085
BE(ecf)	0.563	0.249	5.112	0.024	1.756	1.078–2.861
Lac	0.401	0.312	1.662	0.199	1.493	0.810–2.753

APACHE II, Acute Physiology and Chronic Health Evaluation II; BE, Base Excess; BE(ecf), Base excess of extracellular fluid; IL-6, Interleukin-6; Lac, Lactic Acid; LDH, Lactate Dehydrogenase; PLT, Platelet Count; SOFA, Sequential Organ Failure Assessment; WBC, White Blood Cells.

### 3.4 APACHE II score, LDH, IL-6, oxygenation index, and the predictive ability of BE and BE(ecf) for SAE

**Figure 2:**
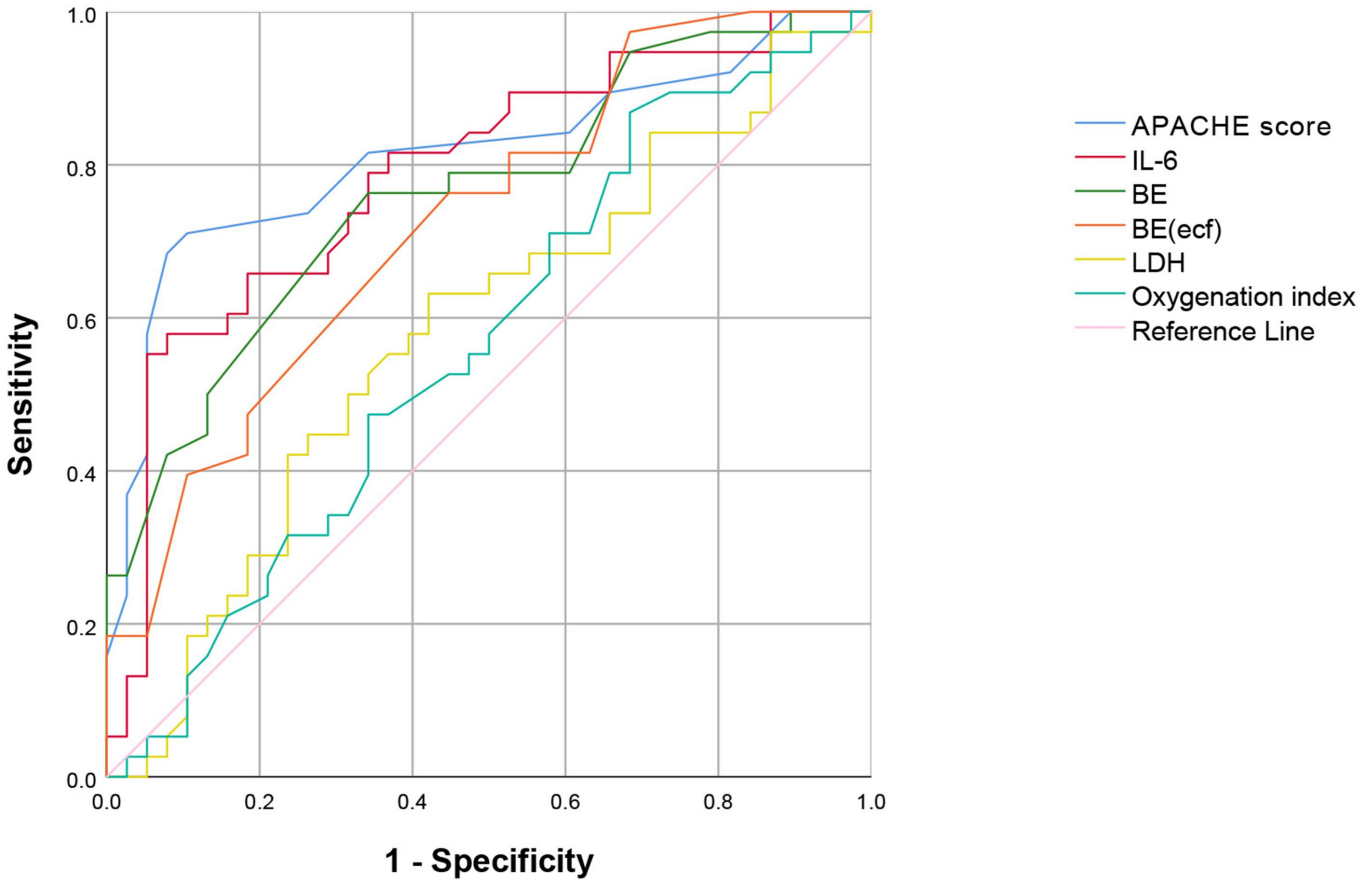
ROC Curve of the APACHE II score, IL-6, BE, BE(ecf), LDH, and oxygenation the prediction of outcome in patients with SAE. ROC Curve, Receiver Operating Characteristic Curve; APACHE II, Acute Physiology and Chronic Health Evaluation II; IL-6, Interleukin 6; BE, Base Excess; BE(ecf), Base Excess (extracellular fluid); LDH, Lactate Dehydrogenase.

The ROC curve analysis indicated that the area under the curve (AUC) for predicting SAE based on the APACHE II score, IL-6, and BE were 0.810 (95% CI: 0.785–0.831), 0.780 (95% CI: 0.743–0.801), and 0.769 (95% CI: 0.730–0.836), respectively (Figure 2). The sensitivity values were 81.1, 77.4, and 70.6%, while the specificity values were 70.3, 72.3, and 71.3%, respectively (Table 4). These findings suggest that the APACHE II score, IL-6, and BE are valuable tools in clinical practice for assessing the risk of SAE in sepsis patients, aiding in early diagnosis and intervention strategies. The results of this study demonstrated that various clinical and biochemical markers hold predictive value for the occurrence of SAE. In contrast, LDH (AUC 0.584) and the oxygenation index (AUC 0.565) were weaker predictors, with lower sensitivity and specificity, suggesting limited utility in predicting SAE (Table 4).

**Table 4:** Predictive efficiency of APACHE II score, LDH, IL-6, oxygenation index, BE, and BE(ecf) for the occurrence of SAE.

Variable	AUC	Optimal truncation value	95% CI	Sensitivity (%)	Specificity degree (%)
APACHE II(points)	0.810	23.5	0.785–0.831	81.10	70.30
LDH(U/L)	0.584	678.5	0.508–0.661	65.30	57.20
IL-6(pg/L)	0.780	2390.8	0.743–0.801	77.40	72.30
Oxygenation index	0.565	183.6	0.536–0.589	66.70	61.80
BE(mmol/L)	0.769	−7.36	0.703–0.836	70.60	71.30
BE(ecf) (mmol/L)	0.723	−8.10	0.649–0.796	69.70	69.71

APACHE II, Acute Physiology and Chronic Health Evaluation II; BE, Base Excess; BE(ecf), Base excess of extracellular fluid; IL-6, Interleukin-6; Lac, Lactic Acid; LDH, Lactate Dehydrogenase.

### 3.5 The clinical efficacy and prognosis in SAE patients

The findings revealed that over the course of 1 to 5 days of treatment, patients in the non-SAE group experienced shorter durations of sedation and quicker recovery compared to those in the SAE group, which required more extensive sedation and analgesia (*p* < 0.05, Table 5). This suggests that patients with SAE have poorer clinical outcomes and a heightened risk of adverse prognoses, underscoring the need for tailored interventions in this population.

**Table 5:** The sedation and analgesia scores after treatment (x̄±s, points) in the SAE and Non-SAE groups.

Groups	Before treatment	After 1 day of treatment	After 2 days of treatment	After 3 days of treatment	After 4 days of treatment	After 5 days of treatment
RASS						
SAE Group	−3.16 ± 0.24	−1.94 ± 0.65	−1.51 ± 0.35	−0.94 ± 0.22	−0.52 ± 0.32	−0.26 ± 0.43
Non-SAE Group	−3.14 ± 0.35	−1.22 ± 0.33	−0.81 ± 0.24	−0.42 ± 0.13	−0.32 ± 0.12	−0.14 ± 0.23
*t*	0.4513	16.25	15.8	11.73	4.513	2.708
*p*	>0.05	<0.05	<0.05	<0.05	<0.05	<0.05
BPS						
SAE Group	8.89 ± 1.11	5.21 ± 0.17	4.12 ± 0.21	3.59 ± 0.23	3.84 ± 0.22	3.85 ± 0.21
Non-SAE group	8.76 ± 0.94	4.51 ± 0.32	3.11 ± 0.32	3.22 ± 0.32	3.54 ± 0.21	3.53 ± 0.42
*t*	1.969	10.6	15.29	5.603	4.543	4.846
*p*	>0.05	<0.05	<0.05	<0.05	<0.05	<0.05

Values are expressed as mean ± standard deviation.

### 3.6 Correlation analysis between RASS score and BPS score and prognosis of sepsis patients

The correlation analysis presented in Table 6 demonstrated a significant relationship between sedation and pain scores and the prognosis of sepsis patients. A negative correlation was observed between the RASS score and prognosis, indicating that higher sedation levels are linked to poorer outcomes (*r* = −0.584, *p* < 0.05). In contrast, the BPS score positively correlated with prognosis, suggesting that higher pain levels are associated with better outcomes (*r* = 0.613, *p* < 0.05).

**Table 6:** Correlation analysis between RASS score and BPS score and prognosis of sepsis patients.

Groups	RASS		BPS	
	*r*	*p*	*r*	*p*
Sepsis	−0.584	<0.05	0.613	<0.05

RASS, Richmond Agitation-Sedation Scale; BPS, Behavioral pain scale.

## 4 Discussion

Sepsis is a critical and life-threatening condition with high morbidity and mortality rates in ICUs globally (23). The immune system becomes dysregulated during sepsis, leading to severe organ dysfunction (24). SAE refers to diffuse brain dysfunction caused by infection, without direct CNS infection or other identifiable causes of brain dysfunction (25). Approximately 70% of patients who suffer from sepsis develop SAE, typically before other organs get involved (26). Clinical symptoms range from mild delirium to severe coma, followed by long-term neurological impairment such as cognitive and memory deficits (27). Consequently, patients experience prolonged hospital stays, along with increased rates of disability and mortality, significantly impacting their prognosis. In recent years, the incidence of SAE in comprehensive ICUs has been as high as 50%, and its incidence and mortality are gradually increasing (28). Therefore, early diagnosis of SAE and analysis of the risk factors of SAE are of great significance for clinicians to take effective measures to prevent and control the occurrence of SAE.

Multivariate logistic regression identified several risk factors for encephalopathy in septic patients, including age, APACHE II score, LDH, IL-6, oxygenation index, BE, and BE(ecf). These factors are likely associated with the significant disruption of vital organ functions, physiological stability, tissue perfusion, and oxygenation in septic patients. This results in elevated SOFA and APACHE II scores, further exacerbating the patient’s condition (29, 30). Furthermore, research has shown that septic patients with higher APACHE II scores are at greater risk of brain tissue damage, increasing their susceptibility to SAE (31). Compared to previous studies (31, 32), our findings align with the growing evidence suggesting that elevated SOFA and APACHE II scores are significant severity indicators in sepsis patients, particularly those developing SAE. In line with the findings of Yang et al. (33), which highlighted a relationship between elevated SOFA scores and poorer clinical outcomes in predicting the 30-day mortality of patients with SAE, our results further emphasize that heightened organ dysfunction is strongly associated with the onset of encephalopathy. SAE is primarily characterized by decreased consciousness, often assessed using the GCS, with delirium being another key symptom. Our findings align with previous studies (28, 34) that reported neurological impairment in patients with sepsis. Sonneville et al. (34) demonstrated that lower GCS scores are associated with longer ICU stays and worse outcomes, with the severity of SAE linked to mortality; specifically, GCS scores between 3 and 8 indicate the highest risk (HR 3.37, 95% CI 2.82–4.03), while even subtle changes in mental status (GCS 13–14) can further increase mortality risk (HR 1.38, 95% CI 1.09–1.38).

IL-6 is a key inflammatory marker that plays a significant role in diagnosing and progressing sepsis by interacting with various cytokines. It is easily detectable and can be an early indicator for diagnosing bacterial infections. Elevated levels of IL-6 can cause neurological damage, contributing to the onset and progression of SAE. The severe inflammatory response in the body leads to changes in blood flow and vascular permeability, impairing oxygen delivery to tissues. Elevated IL-6 levels indicate an acute inflammatory response, commonly seen in sepsis, which can contribute to neuronal injury and dysfunction, potentially leading to SAE. Studies have demonstrated that higher IL-6 concentrations correlate with worse clinical outcomes in septic patients, including increased severity of encephalopathy and higher mortality rates (35). Notably, patients with severe manifestations of SAE, such as coma, exhibit higher plasma concentrations of IL-6 compared to those with milder symptoms like delirium, suggesting a correlation between elevated IL-6 levels and the severity of neurological impairment in sepsis (35, 36).

Although IL-6 is a general indicator of sepsis severity, its specific role as an SAE biomarker remains unclear. Nevertheless, its established significance in assessing sepsis outcomes indicates that monitoring IL-6 levels may offer insights into the prognosis of patients with SAE (35, 36). Our results support these findings, as patients with SAE exhibited significantly higher IL-6 levels than the non-SAE group, suggesting that IL-6 may serve as a valuable marker for assessing the severity of neurological impairment in sepsis. Immune cells rely on glycolysis for energy to maintain normal function under hypoxic conditions, and LDH plays a critical role in this process. LDH can serve as an essential indicator of both the degree of cell necrosis and the body’s immune status. It is present in all tissues and cells of the human body (37). During glycolysis, pyruvate is converted into lactic acid (LA) through the action of LDH, which is essential for the final step of glycolysis (38). In infectious diseases, LDH levels increase, and higher LDH expression is associated with a greater risk of septic-associated encephalopathy (SAE) in sepsis patients.

BE and BE(ecf) are vital indicators of metabolic acidosis (39). In sepsis, uncontrolled infections can trigger shock, tissue hypoxia, and metabolic disruptions, leading to lactic acid accumulation and acidosis. BE and BE(ecf) imbalances are significant in SAE development (2). A negative BE, indicating acidosis, disrupts pH balance, increases cerebral hypoxia, and damages neurons. BE(ecf) imbalances, resulting from fluid shifts due to capillary leakage in sepsis, can cause brain edema, reduced oxygen supply, and electrolyte imbalances (40). Managing BE and BE(ecf) is crucial to reducing the risk of SAE in septic patients. In this study, the incidence of coagulation dysfunction and acid–base imbalance was higher in patients with serious adverse events. The slowing of cerebral blood flow, inadequate cerebrovascular perfusion, and cerebral hypoxia in septic patients with coagulation dysfunction and acid–base imbalance can exacerbate brain damage and increase the risk of septic-associated encephalopathy (41).

This study underscores the pivotal role of sedation and analgesia management in the prognosis of patients with SAE. Sedative agents may exert influence on SAE outcomes through several pathophysiological mechanisms. Specifically, sedation can attenuate sympathetic nervous system hyperactivity, a common feature in sepsis that contributes to systemic inflammation and secondary cerebral injury. By modulating this neurohumoral response, sedatives may confer neuroprotective effects that mitigate neuronal damage. Furthermore, analgesic medications may provide additional neuroprotection by alleviating nociceptive stress and modulating inflammatory pathways, thereby facilitating neurological recovery. The finding that patients with SAE require prolonged durations of sedation and analgesia warrants careful consideration. Prolonged sedation has been implicated in adverse neurological sequelae, including delayed cognitive recovery and increased incidence of delirium. This highlights the critical need to balance adequate sedation to prevent sympathetic overactivation against the potential detrimental effects of oversedation. Implementation of optimized sedation protocols—such as daily sedation interruption, individualized sedation targets, and multimodal analgesic strategies—may contribute to reduced ICU length of stay, enhanced cognitive outcomes, and improved overall prognosis in this population. Given the observed association between SAE and longer ICU stays, lower GCS scores, and poorer clinical outcomes, our findings support the development of tailored sedation and analgesia management protocols informed by SAE risk stratification. Such personalized approaches could enable clinicians to modulate sedation depth and analgesic intensity more precisely, thereby balancing neuroprotection with minimizing sedation-related complications. Ultimately, these insights may inform clinical guidelines aiming to optimize sedation and pain management strategies in critically ill patients with sepsis, to improve neurological and functional outcomes.

This study highlights the diagnostic efficacy of APACHE II score, IL-6, and BE in predicting SAE. The ROC curve analysis revealed AUC values of 0.810, 0.780, and 0.769, respectively, indicating their strong predictive capabilities. The results suggest that the APACHE II score exhibited the highest sensitivity (81.1%) for identifying patients at risk of SAE, enabling early intervention. With sensitivities of 77.4% for IL-6 and 70.6% for BE, both markers are valuable in identifying patients at risk of developing SAE. These results underscore the importance of incorporating IL-6 and BE into clinical assessments alongside the APACHE II score to enhance early detection and management strategies for critically ill patients. These findings align with previous literature identifying APACHE II and IL-6 as critical prognostic indicators in sepsis (31). These findings emphasize the importance of integrating these risk factors into clinical practice to enhance risk stratification, enabling timely interventions and potentially improving outcomes in septic patients. Future research should focus on the clinical integration of these predictive tools to improve the management and care of patients with sepsis.

The significant correlation between RASS and BPS scores and the prognosis of sepsis patients highlights the critical role of these measures in assessing illness severity and predicting outcomes. Our findings align with previous studies reporting a correlation between RASS scores and prognosis, indicating that more profound sedation is linked to poorer outcomes (42). Similarly, the association of higher BPS scores with worse prognoses supports earlier research showing that unaddressed pain in critically ill patients negatively impacts recovery (22). In the present study, the SAE group’s prolonged need for sedation and analgesia suggests a more severe disease course and heightened complication risk (43). These results reinforce the hypothesis that sepsis patients with SAE have poorer prognoses, highlighting the need for early identification and aggressive treatment strategies to improve outcomes. Our findings revealed distinct patterns in sedation (RASS) and analgesia (BPS) scores over the course of treatment, with statistically significant changes observed across the five-day period. These changes likely reflect a complex interaction between patients’ neurological status and clinical management strategies. Specifically, the negative correlation between RASS scores and prognosis suggests that higher RASS scores indicating increased agitation or lighter sedation may be associated with poorer outcomes. This could be due to greater neurological dysfunction or systemic illness severity manifesting as agitation or restlessness. Conversely, the positive correlation observed between BPS scores and prognosis indicates that higher pain scores were linked to better outcomes. This finding may seem counterintuitive but could be explained by improved neurological function in patients who are more able to express pain and more effective pain management protocols implemented in these individuals. Higher BPS scores might also reflect adequate analgesia adjustments responsive to patient discomfort, signifying closer clinical monitoring and intervention. Overall, these results underscore the multifactorial nature of sedation and analgesia management in SAE patients and highlight the need for individualized approaches that consider both neurological recovery and symptom control. Future prospective studies are warranted to elucidate these relationships further and optimize sedation and analgesia protocols in this vulnerable population.

Our study has several limitations that should be considered when interpreting the findings. First, the limited sample size may restrict the generalizability of the results and reduce the statistical power to detect significant associations. Second, as a retrospective analysis, the study is subject to potential biases, such as selection bias and inaccuracies in data recording. Additionally, the study did not examine all potential risk factors for septic-associated encephalopathy, which may introduce bias and limit the comprehensiveness of the findings. Third, there is a lack of detailed information regarding the exact time elapsed since the onset of sepsis for each patient. Although we included clinical data collected within 24 h of ICU admission, heterogeneity in the timing of sepsis onset prior to admission may have influenced the patients’ inflammatory status at the time of data collection. This variability could affect the interpretation of inflammation-related biomarkers and their association with clinical outcomes. Fourth, differences in clinical practices, such as sedation protocols and analgesic use, can influence outcomes and further limit the findings’ generalizability. Finally, although sedation and analgesia scores (RASS and BPS) were assessed by trained ICU staff following standardized protocols, variability inherent in clinical practice and differences in individual clinician assessment may have introduced measurement bias. Moreover, while sedation and pain management adhered to institutional guidelines consistent with national standards, individualization of sedation targets and analgesia strategies based on patient condition could have influenced score variability and, consequently, study outcomes. These factors should be considered when interpreting the associations observed between sedation/analgesia scores and SAE prognosis. Therefore, larger multicenter studies with more comprehensive data collection and standardized methodologies are needed to better understand SAE’s risk factors and predictors.

## 5 Conclusion

Our study elucidated the impact of sedation and analgesia scores on critically ill patients with SAE, identifying key risk factors such as age, APACHE II score, LDH, IL-6, BE, and BE(ecf), all linked to organ dysfunction and poor outcomes. Moreover, significant correlations between RASS and BPS scores and patient prognosis underline their importance in assessing illness severity and guiding treatment decisions. Future research should focus on the specific contributors to SAE, including underlying infections and immune responses, to develop targeted management strategies. Comprehensive clinical interventions, including timely recognition of sepsis and effective management of sedation and analgesia, are crucial for reducing the incidence of SAE and enhancing patient outcomes.

## Data Availability

The original contributions presented in the study are included in the article/supplementary material, further inquiries can be directed to the corresponding author.
